# Comprehensive Transcriptome and Pathway Analyses Revealed Central Role for Fascin in Promoting Triple-Negative Breast Cancer Progression

**DOI:** 10.3390/ph14121228

**Published:** 2021-11-26

**Authors:** Rayanah Barnawi, Samiyah Al-Khaldi, Salma Majid, Amal Qattan, Tala Bakheet, Mohannad Fallatah, Hazem Ghebeh, Nehad M. Alajez, Monther Al-Alwan

**Affiliations:** 1 Stem Cell and Tissue Re-Engineering Program, King Faisal Specialist Hospital and Research Centre, Riyadh 11211, Saudi Arabia; brayyanah@kfshrc.edu.sa (R.B.); hghebeh@kfshrc.edu.sa (H.G.); 2 National Center for Biotechnology, Life Science and Environment Research Institute, King Abdulaziz City for Sciences and Technology, Riyadh 12354, Saudi Arabia; salkhaldi@kacst.edu.sa (S.A.-K.); mfallatah@kacst.edu.sa (M.F.); 3 National Institute on Alcohol Abuse and Alcoholism (NIAAA), National Institutes of Health (NIH), Bethesda, MD 20892, USA; majidsn@nih.gov; 4 Molecular Oncology, King Faisal Specialist Hospital and Research Centre, Riyadh 11564, Saudi Arabia; akattan@kfshrc.edu.sa; 5 Molecular Biomedicine Program, King Faisal Specialist Hospital and Research Centre, Riyadh 11564, Saudi Arabia; tala@kfshrc.edu.sa; 6 College of Medicine, Al-Faisal University, Riyadh 11533, Saudi Arabia; 7 Translational Cancer and Immunity Center, Qatar Biomedical Research Institute, Hamad Bin Khalifa University, Qatar Foundation, Doha P.O. Box 34110, Qatar; 8 College of Health & Life Sciences, Hamad Bin Khalifa University, Qatar Foundation, Doha P.O. Box 34110, Qatar

**Keywords:** fascin, breast cancer, miRNA, pathway analysis, transcriptome, IPA

## Abstract

Recent years have witnessed major progress in development of novel therapeutic agents such as chemotherapy, targeted therapy and immune checkpoint inhibitors for breast cancer. However, cancer-related death remains high especially in triple-negative breast cancer (TNBC) due limited therapeutic options. Development of targeted therapies for TNBC requires better understanding of biology and signaling networks that promote disease progression. Fascin, an actin bundling protein, was identified as a key regulator of many signaling pathways that contribute to breast cancer progression. Herein, fascin ShRNA was used to generate stable fascin knockdown (FSCN1^KD^) in the MDA-MB-231 TNBC cell line and then were subjected to comprehensive mRNA and miRNA transcriptome analysis. We identified 129 upregulated and 114 downregulated mRNA transcripts, while 14 miRNAs were differentially expressed in FSCN1^KD^. Ingenuity pathway analysis (IPA) was used to predict the impact of differentially expressed transcripts on signaling pathways and functional categories and to construct miRNA-mRNA regulatory networks in the context of FSCN1 knockdown. Compared to FSCN1^KD^, fascin-positive (FSCN1^CON^) breast cancer cells showed enrichment in genes promoting cellular proliferation, migration, survival, DNA replication and repair. Expression of FSCN1^high^ (identified in BRCA dataset from TCGA) in conjunction with elevated expression of the top 10 upregulated or decreased expression of the top 10 downregulated genes (identified in our FSCN1^CON^ vs. FSCN1^KD^) correlates with worst survival outcome. Taken together, these data confirmed fascin’s role in promoting TNBC progression, and identified a novel opportunity for therapeutic interventions via targeting those FSCN1-related transcripts.

## 1. Introduction

Breast cancer is the most common cancer in women worldwide and is the leading cause of cancer-related mortality in women. Overall and disease-free survival of breast cancer patients were both improved by the early detection and introduction of targeted therapy and immune checkpoint inhibitors in combination with conventional treatment modalities [1]. Nonetheless, breast cancer-related death remains high mainly due to drug resistance and metastasis [2], thus stressing the need for better understanding of breast cancer biology to develop more effective targeted therapies. Triple-negative breast cancer (TNBC) accounts for 12–20% of all breast cancer subtypes [3]. Compared to other breast cancer subtypes, TNBC has the most aggressive behavior with high recurrence rate and worst outcome [4]. This has been mainly attributed to limited therapeutic options due to lack of biomarkers or valid treatment targets. Therefore, identification of biomarkers or signaling pathways that are differentially enriched in TNBC could potentially expand our knowledge and accelerate therapeutic development with the aim to hinder or slow the disease progression.

Fascin is an actin bundling protein with high expression in the filopodia, critical cell protrusions at the leading edge of migrating cells [5]. Moreover, it was reported to regulate adhesion and filopodia formation in migrating cancer cells [6]. Inhibition of fascin using RNA interference reduced the number of filopodia and disrupted the organization of the actin bundles [7,8]. Fascin expression is induced in many transformed epithelial cells including breast, and its expression level is considered a biomarker of worst clinical outcome [9,10,11,12]. Fascin expression was reported in high percentage (87.8%) of TNBC as compared to the other breast cancer subtypes [13] and thus has been proposed as a diagnostic marker for TNBC and potential therapeutic target [13,14,15]. Most importantly, fascin inhibitors were shown to block TNBC metastasis in tumor metastasis mouse model [16,17,18] and to reduce the growth of specific TNBC, which express high levels of epidermal growth factor receptor [18,19]. We have previously reported a crucial role for fascin in regulating drug resistance [20] and metastasis [9] and have identified several underlying mechanisms that have contributed in this process [21,22,23]. More inclusive studies are needed to identify enriched molecular signature in fascin-positive TNBC for potential therapeutic targeting. 

Comprehensive transcriptomic studies that compare differentially expressed transcripts upon manipulation of fascin expression in TNBC is expected to expand our understanding of the underlying mechanisms of fascin contribution to breast cancer progression, which eventually will stimulate therapeutic target development. Herein, we carried a comprehensive transcriptome analysis on fascin knockdown TNBC cell line (MDA-MB-231) in comparison to their fascin-expressing counterparts. Results from our fascin knockdown cells showed enrichment of genes that promoted disease progression in fascin-positive breast cancer cells. Collectively, this study provides more comprehensive understanding of the oncogenic role of fascin and its effectors, which might represent valuable tumor biomarkers and therapeutic targets for TNBC. Therefore, targeting of signaling pathways that are enriched in fascin-positive breast cancer may provide a novel therapeutic window for halting the disease progression, especially in light of the limited therapeutic options for TNBC.

## 2. Results

### 2.1. FSCN1 Is Enriched with Prognostic Markers of Poor Survival

**Figure 1:**
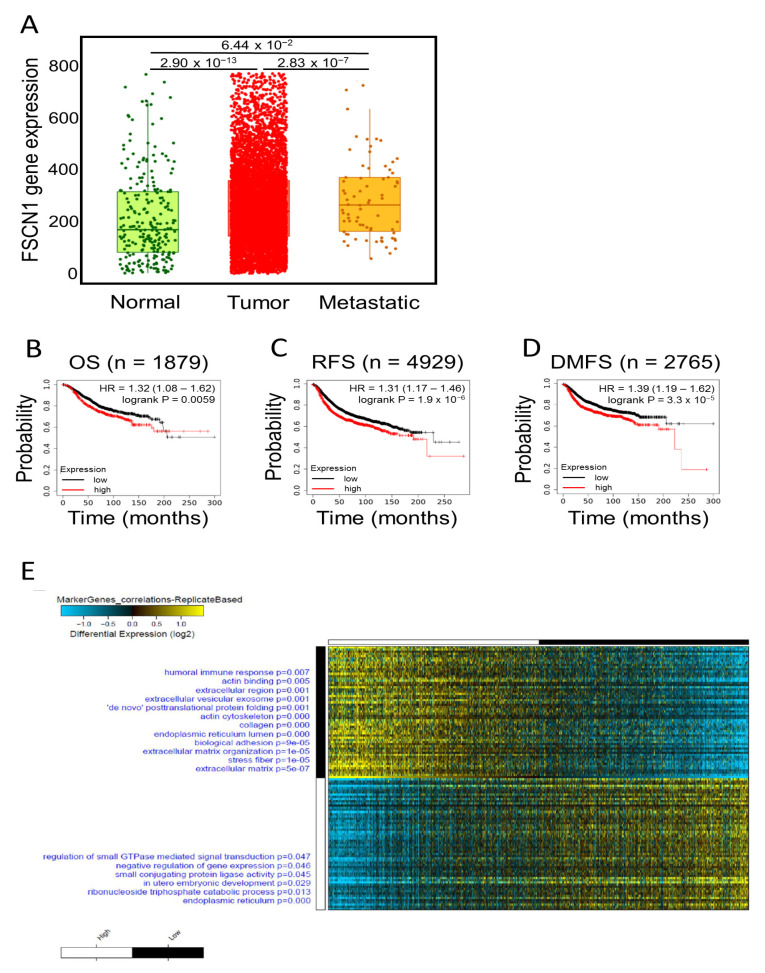
Expression, survival and differential gene expression in FSCN1^high^ compared to FSCN1^low^ breast cancer patients from TCGA dataset. (**A**) Boxplot showing fascin expression in normal (*n* = 242), tumor (*n* = 7569) and metastatic (*n* = 82) breast cancer patients. Kaplan-Meier plots showing (**B**) overall survival (OS; *n* = 1879), (**C**) recurrence-free survival (RFS; *n* = 4929) and (**D**) distant metastasis-free survival (DMFS; *n* = 2765) as function of median FSCN1 expression in breast cancer patients. The significance between FSCN1^high^ and FSCN1^low^ groups was calculated using the log-rank test. *p*-values are indicated on each plot. (**E**) Marker discovery analysis to identify putative markers selectively expressed in FSCN1^high^ (*n* = 534) vs. FSCN1^low^ (*n* = 545) stratified according to median FSCN1 expression from TCGA BRCA dataset (*n* = 1217). Enriched gene ontology (GO) associations are indicated on the y axis.

We have previously reported a critical role for fascin in regulating breast cancer chemoresistance, resulting in poor survival [20]. Subsequently, we delineated some underlying mechanism that promote fascin contribution in poor survival [21,22]. Here we aimed to define novel genes that are enriched in fascin-positive breast cancer cells and contribute in the disease progression. A large cohort of breast cancer patients from the publicly available Kaplan–Meier plotter portal dataset [24] was used to assess fascin expression and its impact on overall survival (OS; *n* = 1879), recurrence-free survival (RFS; *n* = 4929) and distant metastasis-free survival (DMFS; *n* = 2764). Remarkable increase in fascin expression was observed in the breast cancer cohort (*n* = 7569) compared to samples from the normal group (*n* = 242) (Figure 1A). Furthermore, metastatic patients (*n* = 82) exhibited higher expression of fascin as compared to the normal group. Concordantly, FSCN1^high^ breast cancer patients showed the worst OS, RFS and DMFS as compared to FSCN1^low^ group (Figure 1B–D). Importantly, the power of significance (*p* value) between FSCN1^high^ and FSCN1^low^ groups increased from 0.0059 in OS to 1.9 × 10^−6^ and 3.3 × 10^−5^ for RFS and DMFS, respectively. These data emphasize the role of fascin not only in predicting the OS, but its contribution to RFS and DMFS, making it useful biomarker for assessing the prognosis of the disease.

**Figure 2:**
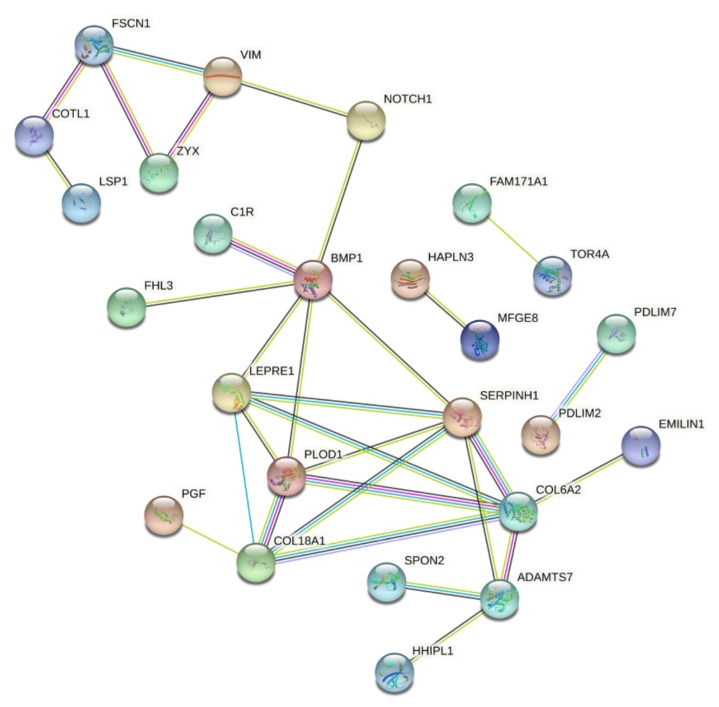
Protein-protein interaction network in FSCN1^high^ compared to FSCN1^low^ BRCA. Constructed protein-protein interaction network of 60 enriched genes in FSCN1^high^ (*n* = 534) vs. FSCN1^low^ (*n* = 545) breast cancer patients from TCGA dataset. Nodes represent proteins and different line intensities denote the type of evidence for the interaction. Statistical analysis results for the network: number of nodes: 59, number of edges: 28; average node degree: 0.94; average local clustering coefficient: 0.26; expected number of edges: 7; PPI enrichment *p*-value = 5.7–10.

To identify putative markers that are selectively associated with high fascin expression, we have used the publicly available breast cancer patients’ gene expression dataset from The Cancer Genome Atlas (TCGA; *n* = 1217). These data were subjected to marker discovery analysis comparing FSCN1^high^ (*n* = 534) vs. FSCN1^low^ (*n* = 545). FSCN1^high^ group showed enrichment in genes that are involved in regulating many cellular processes such as the extracellular matrix organization, stress fibers, biological adhesion, actin binding and posttranslational protein folding (Figure 1E). On the contrary, FSCN1^low^ group showed enrichment of genes that are involved in regulation of other cellular process such as small GTPase mediated signal transduction, negative regulation of gene expression and endoplasmic reticulum. The top 60 enriched genes in FSCN1^high^ vs. FSCN1^low^ breast cancer patients from TCGA dataset were then subjected for network analysis using AltAnalyze software to construct protein-protein interaction. These data showed nodes with evidence of protein-protein interaction (Figure 2). Some of the enriched (such as VIM and NOTCH1) and reduced (such as ESR) genes in FSCN1^high^ groups are consistent with our previous results [9,23]. These data establish that the level of fascin expression in breast cancer samples is associated with differentially expressed genes that regulate key cellular process and promote disease progression. 

### 2.2. Differential Genes Enrichment in FSCN1 Breast Cancer Cells

**Figure 3:**
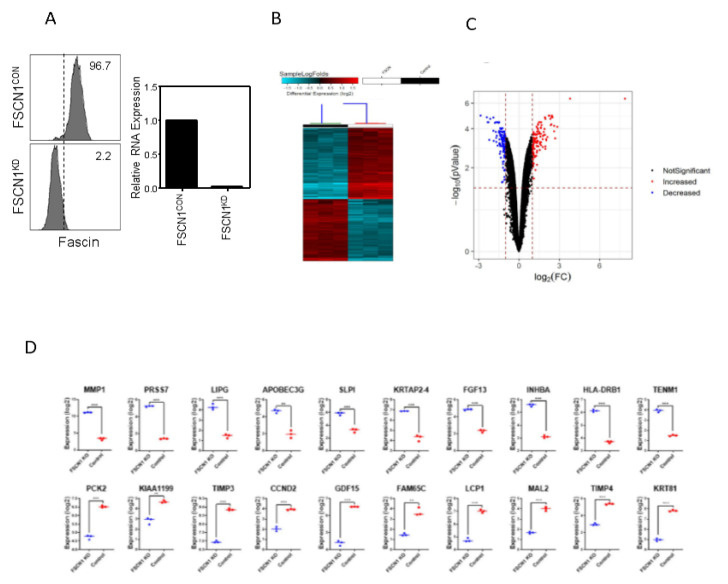
Differential gene expression analysis on FSCN1^KD^ compared to FSCN1^CON^ MDA-MB-231 cells. (**A**) Left: FACS histograms showing the levels of fascin expression in FSCN1^CON^ and FSCN1^KD^ MDA-MB-231 cells. Right: Bar graph showing the relative RNA expression of fascin in FSCN1^CON^ and FSCN1^KD^ MDA-MB-231 cells. (**B**) Heatmap depicting the relative expression levels of differentially expressed genes in FSCN1^KD^ compared to FSCN1^CON^ MDA-MB-231 cells. Each column represents one replica, and each row represents a single transcript. The expression level of each transcript in a single sample is depicted according to the color scale. (**C**) Volcano plot representation of significantly altered genes in FSCN1^KD^ compared to FSCN1^CON^ MDA-MB-231 cells. Red and blue colors indicate the genes with significantly increased or decreased expression, respectively. Black color indicates no significant change. (**D**) Expression of top 10 upregulated and top 10 downregulated transcripts in FSCN1^KD^ compared to FSCN1^CON^ MDA-MB-231 cells based on microarray data (*n* = 3). ** *p* < 0.005, *** *p* < 0.0005.

To provide mechanistic insight into the role of FSCN1 in promoting TNBC progression, stable fascin knockdown MDA-MB-231 TNBC model [23] was used to characterize the alterations in gene expression and signaling networks. Fascin-positive (FSCN1^CON^) and -negative (FSCN1^KD^) cells (Figure 3A) were subjected for comparative transcriptome analysis. Hierarchical clustering and volcano plot based on differentially expressed RNA transcripts showed clear clustering of (FSCN1^CON^) from (FSCN1^KD^) breast cancer cells (Figure 3B,C).

**Figure 4:**
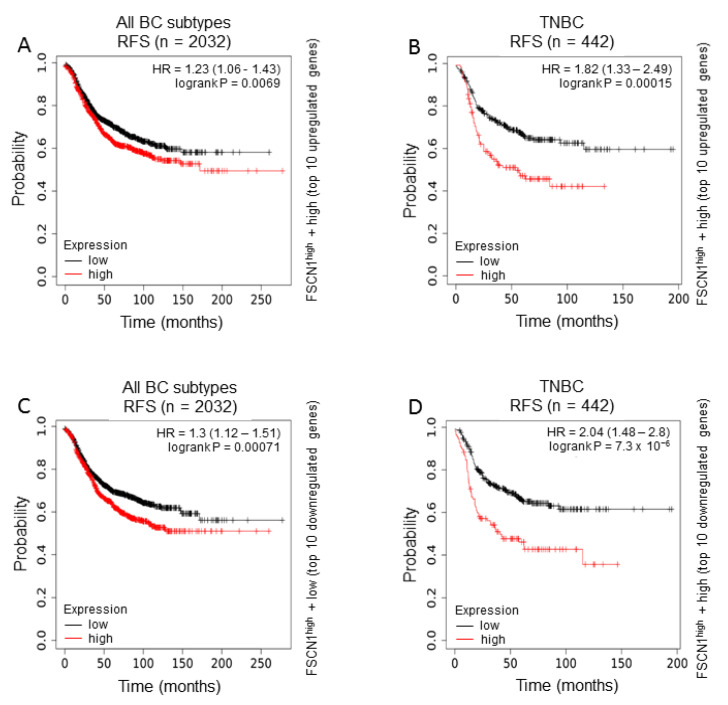
Effect of the differentially identified genes in MDA-MB-231 breast cancer cells on survival of FSCN1^high^ compared to FSCN1^low^ breast cancer patients from TCGA dataset. Kaplan-Meier plots from TCGA dataset showing survival of (**A**,**C**) all breast cancer subtypes (RFS; *n* = 2032) or (**B**,**D**) TNBC subtype (RFS; *n* = 442). Survival of FSCN1^high^ in conjunction with (**A**,**B**) high expression of the top 10 upregulated genes or (**C**,**D**) low expression of the top 10 downregulated genes identified in FSCN1^CON^ MDA-MB-231 cells. The significance between FSCN1^high^ and FSCN1^low^ groups was calculated using the log-rank test. *p*-values are indicated on each plot.

Based on −2.0 ≥ FC ≥ 2.0 with significant value (*p* < 0.05), 129 upregulated and 114 downregulated transcripts were identified (Table S1). Figure 3D shows expression of top 10 upregulated and top 10 downregulated transcripts in FSCN1^KD^ compared to FSCN1^CON^ MDA-MB-231 cells based on microarray data. We subsequently used TCGA dataset to assess the prognostic value of FSCN1^KD^-derived gene signature on the survival of all breast cancer subtypes or TNBC patients. Interestingly, FSCN1^high^ in conjunction with high expression of the top 10 upregulated genes (identified in our FSCN1^CON^ MDA-MB-231 cells) significantly correlates with poor RFS survival in all breast cancer subtypes (Figure 4A).

This correlation become more pronounced in TNBC subtype than all subtypes combined as reflected by the increase in hazard ratio from 1.23 to 1.82 and the power of significance (*p* value) from 0.0069 to 0.00015 (Figure 4B). Similarly, FSCN1^high^ in conjunction with low expression of the top 10 downregulated genes (identified in our FSCN1^CON^ MDA-MB-231 cells) significantly correlates with poor RFS survival in all breast cancer subtypes (Figure 4C). This correlation become more pronounced in TNBC subtype than all subtypes combined as reflected by the increase in hazard ratio from 1.3 to 2.04 and the power of significance (*p* value) from 0.00071 to 7.3 × 10^−6^ (Figure 4D). The poor RFS of FSCN1^high^ patients with the differentially expressed genes that become more significant in TNBC subtype demonstrated a clinical relevance to our in vitro findings. Collectively, these data suggest that fascin co-expression with these genes could serve as prognostic biomarkers for breast cancer. 

### 2.3. Mechanistic Network Analysis Predicts Activation of Cell Proliferation and Survival Networks in FSCN1^CON^ Breast Cancer

**Figure 5:**
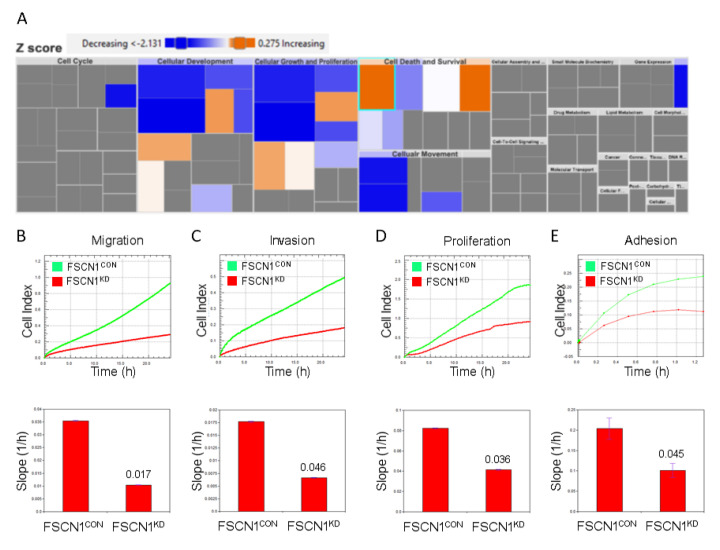

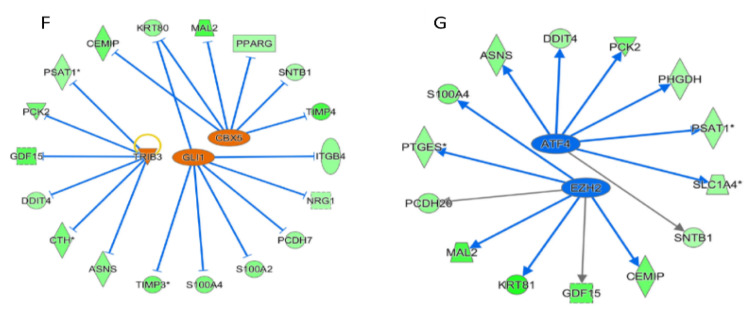
Effector analysis of differentially regulated gene transcripts in FSCN1^KD^ MDA-MB-231 breast cancer cells. (**A**) Tree map (hierarchical heat map) depicting affected functional categories based on differentially expressed genes in FSCN1^KD^ compared to FSCN1^CON^ MDA-MB-231 cells where the major boxes represent a category of diseases and functions. Each colored rectangle is a particular biological function or disease and the color range indicates its predicted activation state—increasing (orange) or decreasing (blue). Darker colors indicate higher absolute Z-scores. In this default view, the size of the rectangles is correlated with increasing overlap significance. Live cell assays showing migration (**B**), invasion (**C**), proliferation (**D**) and adhesion to collagen IV (**E**) in FSCN1^KD^ compared to FSCN1^CON^ MDA-MB-231 cells. Results in B-E are the mean of triplicates ± SD and are representative of three independent experiments. Upstream regulator analysis depicted activated GLI1, CBX5 and TRIB3 (**F**) and suppressed EZH2 and ATF4 (**G**) networks based on IPA analysis of data in Table S1.

To gain insight into the putative effect of fascin clustering with the differentially expressed genes on downstream biological activities and functions, we have subjected differentially expressed genes in FSCN1^CON^ vs. FSCN1^KD^ to IPA analysis. Figure 5A showed rectangles with different color code, dimension and intensity that indicate a family of associated biological functions or diseases. The rectangle dimension indicates the associated functions that are predicted to be most significantly affected. The color intensity indicates higher absolute Z-scores, where blue and orange reflects decrease and increase, respectively. Functional categories associated with cellular movement and invasion as well as cell growth and proliferation were enriched, while those associated with cellular development as well as cell death and survival were diminished in FSCN1^CON^ cells. In agreement with those data, in vitro live assays showed reduced migration, invasion, proliferation, and adhesion in our FSCN1^KD^ breast cancer cells (Figure 5B–E). Further upstream regulator analysis using IPA showed activated GLI1, CBX5 and TRIB3 (Figure 5F) and suppressed EZH2 and ATF4 (Figure 5G) networks in FSCN1^KD^ breast cancer cells. Collectively, these data demonstrated association between the differentially regulated transcripts in FSCN1^KD^ compared to FSCN1^CON^ MDA-MB-231 cells and increase in cell proliferation, migration, while the cell death-associated functional categories were suppressed.

### 2.4. Predicated Protein-Protein Interaction Network in FSCN1^CON^ Breast Cancer

**Figure 6:**
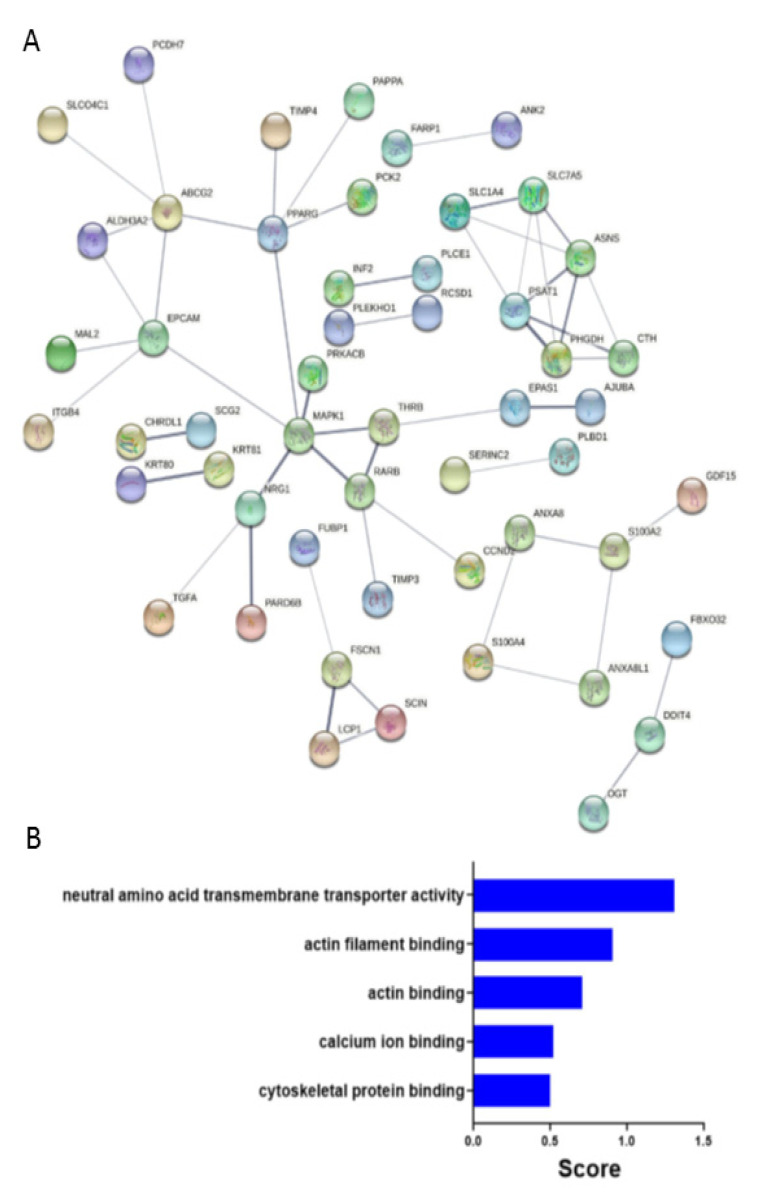
Protein-protein interaction network and gene ontology (GO) enrichment. (**A**) Constructed protein-protein interaction network of 93 downregulated genes in FSCN1^KD^ compared to FSCN1^CON^ MDA-MB-231 cells. Nodes represent proteins and different line intensities denote the type of evidence for the interaction. Statistical analysis results for the network: number of nodes: 88, number of edges: 49; average node degree: 1.1; average local clustering coefficient: 0.44; expected number of edges: 31; PPI enrichment *p*-value =0.001. (**B**) GO enrichment bar chart of downregulated genes in FSCN1^KD^ compared to FSCN1^CON^ MDA-MB-231 cells showing most enriched GO terms.

We subjected the 93 downregulated genes in FSCN1^KD^ compared to FSCN1^CON^ MDA-MB-231 cells to construct protein-protein interaction network using STRING. Result analysis of the network based on statistical difference identified 88 nodes (Figure 6A).

MAPK1, EPCAM, and ABCG2 are examples of some of the key nodes that were identified and are found to be relevant for the disease progression [25,26,27]. Indeed, gene ontology enrichment bar chart of downregulated genes in FSCN1^KD^ compared to FSCN1^CON^ MDA-MB-231 cells showing most enriched GO categories that are related to neutral amino acid transmembrane transport activity, actin and cytoskeletal binding, and calcium ion binding (Figure 6B). Altogether, these data suggest an active role for fascin in regulating a number of key PPI networks and functional categories promoting tumorigenesis.

**Figure 7:**
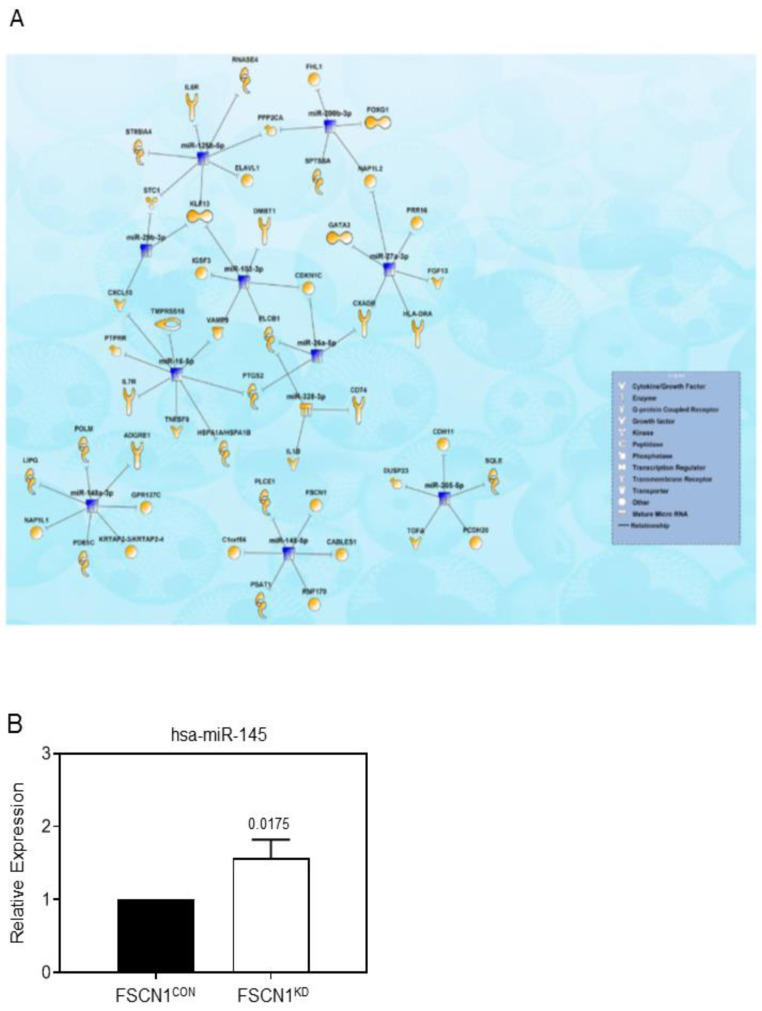
Network Analysis illustrating the interactions between differentially expressed miRNAs and mRNAs in FSCN1^KD^ MDA-MB-231 breast cancer cells. (**A**) Network illustrating the interaction between differentially expressed miRNAs and mRNAs in FSCN1^KD^ MDA-MB-231 based on IPA microRNA target filter analysis. (**B**) Bar graph showing relative RNA expression of hsa-miR-145 in FSCN1^KD^ compared to FSCN1^CON^ MDA-MB-231 cells.

To gain more insight into the molecular mechanisms by which fascin regulates breast cancer progression, we performed microRNA array analysis of FSCN1^KD^ compared to FSCN1^CON^ MDA-MB-231 cells. We identified 2 upregulated and 12 downregulated miRNAs (2.0-fold change, *p*  <  0.05, Table S2) in FSCN1^KD^ cells, which subsequently were subjected to IPA microRNA target filter analysis utilizing both miRNA and mRNA data. Data presented in Figure 7A illustrates miRNA-mRNA networks based on transcriptomic data from the FSCN1^KD^ and FSCN1^CON^ models. The interaction network is illustrated as mature miRNA (presented as nodes) and biological relationships, which are displayed using different shapes representing the functional class of the target gene as illustrated in the corresponding legend (Figure 7A). We have confirmed the differential expression of hsa-miR-145 miRNA following fascin knockdown in our MDA-MB-231 breast cancer cells. FSCN1^KD^ cells express increased levels of hsa-miR-145, indicating an inverse relationship between hsa-miR-145 and FSCN1 expression (Figure 7B). In addition to the hsa-miR-145-FSCN1 network, IPA analysis revealed regulation of several genes belonging to different functional categories, including cytokines, enzymes, growth factors, transporters, transcription regulators. Altogether, these differentially regulated miRNAs are indicative of breast cancer-related functional categories with more confidence and high level of predicted relationship.

## 3. Discussion

Breast cancer is a heterogeneous disease that can be classified into four major subtypes: luminal A, luminal B, HER+, and TNBC. Luminal subtypes, especially luminal A, have the best prognosis and survival outcome mainly due to the availability of endocrine therapies [28]. On the contrary, TNBC are the most aggressive subtype with the worst survival outcome mainly due limited alternative therapeutic options [29,30]. In this study, we identified the transcriptional landscape enriched in TNBC expressing high fascin, which is associated with shorter survival of FSCN1^high^ patients particularly the TNBC subtype, demonstrating a clinical relevance of our in vitro findings in this breast cancer subtype.

Fascin knockdown in MDA-MB-231 TNBC model reveal a number of differentially expressed transcripts (129 upregulated and 114 downregulated). Some of the enriched transcripts in our FSCN1^CON^ breast cancer cells are in high concordances with those that are found in FSCN1^high^ group of the publicly available TCGA database. The fact that proteins identified based on differential gene expression from TCGA dataset are from breast cancer patients, which include various cell types, while those identified from FSCN1^KD^ are from cell line data may explain this inconsistency between the two lists. The decrease in ESR and the increase in NOTCH1 and VIM that was observed in FSCN1^high^ group of BCRA cohort from the TCGA database are in line with our previous results, which demonstrated promotion of NOTCH1 and VIM by fascin [9,23]. Most importantly, the association between the differentially expressed genes in FSCN1^high^ and increase in functional categories related to cellular movement, invasion, growth and proliferation as well as decrease in those related to cellular development, death and survival is consistent with oncogenic roles of fascin that were demonstrated in previous studies [9,21,22,23].

The finding of increased expression of KIAA1199 [31], GDF15 [32] and KRT81 [33] in our FSCN1^CON^ compared to FSCN1^KD^ TNBC is consistent with its over-expression in this breast cancer subtype and promotion of their migration. Enrichment of CCND2 in TNBC especially post neoadjuvant chemotherapy [34] is consistent with its increased expression in our FSCN1^CON^. Similarly, the over-expression of MAL2 in TNBC and its contribution to immune evasion and poorer survival [35] supports our finding of increased expression of MAL2 in FSCN1^CON^. The elevated level of TIMP4 in early stage estrogen receptor-negative breast cancer and association with poorer clinical outcome [36] is consistent with the finding of increased expression of TIMP4 in FSCN1^CON^. On the other hand, the decreased expression of APOBEC3G in our FSCN1^CON^ compared to FSCN1^KD^ is consistent with positive correlation between its increased expression and favorable prognosis in TNBC [37]. In addition, the decreased expression of KRTAP2-4 in FSCN1^CON^ is consistent with its downregulation by miRNA-200c in TNBC to promote metastasis and reduce survival [38]. There is association between high expression of HLA-DRB1 with increased tumor-infiltrating lymphocytes and therapeutic response in TNBC [39] consistent with its reduced expression in our FSCN1^CON^. No known function for TENM-1 in TNBC, but if it serves a tumor suppressor role like what has been suggested for TENM2 in various type of tumors [40] then that is consistent with the decreased expression in our FSCN1^CON^. To large extent, our transcriptome data fit well with the oncogenic role of fascin in TNBC, further supports its use as a prognostic marker in this subtype and shed light on the pathway that could be used for therapeutic targeting.

Deregulated expression of miRNAs has been implicated in regulating many diseases including cancer. For example, miRNA-145 is under-expressed in various tumors including breast and is considered to be a tumor suppressor gene due to its inhibitory effect on tumor cell proliferation, invasion, and metastasis [41]. High miRNA-145 expression has also been reported to increase tumor cell sensitivity to chemotherapy. Importantly, miRNA-145 was reported to target fascin and inhibit metastasis or invasion in many cancer types including colorectal [42], nasopharyngeal [43], gastric [44] and non-small-cell lung cancer [45]. Our findings of repressed miRNA-145, increased migration, invasion, proliferation and adhesion in our FSCN1^CON^ TNBC may explain the poor prognosis and shorter survival. The expression level of miRNA-145 following treatment of TNBC with fascin inhibitors has not been assessed [16,17,18], which could validate the miRNA-145 repression by fascin. miRNA-145 was also predicted to regulate several other genes in our FSCN1^KD^ model. Similarly, the reduced expression of miRNA-205 in our FSCN1^CON^ TNBC is consistent with previous study, where its upregulation was reported to inhibit proliferation, cell cycle progression and tumor growth [46]. The increase expression of the onco-suppressor miRNA-424 in our FSCN1^CON^ TNBC is consistent with its association with aggressive state including advanced clinical stage, tumor size and distance metastases [47]. The increase expression of miRNA-200c in our FSCN1^CON^ TNBC is consistent with its role in promoting migration and invasiveness of TNBC [48]. Moreover, the increase expression of miRNA-27b in our fascin expressing TNBC is consistent with its role in regulating migration and association with poor clinical outcome [49]. Of the differentially regulated miRNAs upon fascin knockdown in TNBC, miRNA-145, miRNA-205, miRNA-424, miRNA-200c, miRNA-27b may explain the aggressive behavior of fascin-expressing TNBC. The remaining identified miRNA-mRNA networks in the FSCN1^KD^ model and their plausible role in driving TNBC progression warrants further investigation.

We previously have delineated many of the underlying mechanisms of fascin regulation of key signaling pathways that promote cancer progression such as AKT, NF-kB, [9], FAK [20], NOTCH1 [23], ITGB1 [22] and CTNNB [21]. However, fascin involvement in regulating many other signaling pathways remained largely unexplored and the results reported in this study should stimulate further investigations toward this direction. For example, one could speculate that fascin, via its actin bundling activity, may act as scaffold to promote or suppress complex formation between interacting partners. While the exact mechanism of fascin regulation of the differentially expressed transcripts in this study are not elucidated, these findings shed lights on the oncogenic effect of fascin in promoting disease progression particularly in TNBC via modulating other biomarkers and signaling pathways. Therefore, targeting fascin or its associated network may present a novel therapeutic window as single or in combination with other treatment modalities for better clinical outcome.

## 4. Materials and Methods

### 4.1. Cells

The human triple-negative breast cancer (TNBC) cell line MDA-MB-231 (HTB-26) was purchased from ATCC (Manassas, VA, USA) and were maintained in DMEM supplemented with 10% fetal bovine serum (Invitrogen, Paisley, UK), 200 mM L-glutamine (Invitrogen) and antibiotic-antimycotic liquid (Invitrogen) at 37 °C in a 5% CO2 humidified atmosphere.

### 4.2. Fascin Knockdown

Stable fascin knockdown in MDA-MB-231 cells were generated as previously described [9]. Briefly, lentiviral vectors (Thermo Scientific, Paisley, UK) expressing either fascin shRNA (clone Id: TRCN0000123039; sequence TTCCAGTTTGAAAGGCAAGGG) or scrambled shRNA were used to generate fascin knockdown (FSCN1^KD^) or control (FSCN1^CON^) MDA-MB-231 cells, respectively. Fascin knockdown was confirmed using flow cytometery and western blot.

### 4.3. Differential Gene Expression Analysis

Total RNA was extracted from FSCN1^KD^ or FSCN1^CON^ cells using QIAGEN RNeasy Mini Kit (QIAGEN, Valencia, CA, USA) following the manufacturer’s instructions. Integrity of the extracted RNA was determined using the Agilent Bioanalyzer 2100 system (Agilent Technologies, Santa Clara, CA, USA) before the cDNA synthesis using High Capacity RNA-to-cDNA Kit (Applied Biosystems, Paisley, UK). The cDNA was then subjected for global gene transcription using GeneChip (Affymetrix, Santa Clara, CA, USA), where generated fluorescent oligonucleotide probes were hybridized to the GeneChips^®^ Human Genome HG-U133 Array in a GeneChip^®^ hybridization oven as per the manufacturer’s instructions. This array has around 55,000 probe sets, which represent over 39,000 transcripts from 33,000 previously identified human genes. Gene Array scanner (Affymetrix) was used for visualization and GeneChip^®^ Operating Software was utilized for image quantitation.

### 4.4. Microarray Data Analysis

Microarray expression data from FSCN1^KD^ vs FSCN1^CON^ MDA-MB-231 cells were then subjected to quantile normalization and differential analysis and hierarchical clustering as described before [50]. Transcripts exhibiting −2.0 ≥ FC ≥ 2.0 and *p* < 0.05 were considered significant and were used for Ingenuity Pathway Analysis (IPA) analysis. Volcano plots were generated using R studio Version 1.2.5001.

### 4.5. qRT-PCR for Mature MicroRNAs and Bioinformatics Analysis

Total RNA was isolated from FSCN1^KD^ vs FSCN1^CON^ MDA-MB-231 cells as above and qRT-PCR for mature miRNA was run using miScript miRNA PCR Array Human Breast Cancer-MIHS-109Z (Qiagen, Hilden, Germany) as previously described [51]. This miRNA PCR Array Panel contains 84 mature miRNAs that are most relevant to breast cancer tumorigenesis and all qPCR reactions were run in duplicate. The relative expression of a given miRNA was assessed by computing 2(−ddCt) (2(−ΔΔCt)). The miRNA targets and the biological pathways they were involved in were predicted using IPA microRNA filter as previously described [52].

### 4.6. Ingenuity Pathways Analysis (IPA)

Differentially expressed genes from the microarray data (2.0 FC, *p* < 0.05 Adj) were imported into the IPA Software (Ingenuity Systems Inc., Germantown, MD, USA) as we previously described [53]. Functional regulatory networks and canonical pathways were determined using upstream regulator analysis (URA), downstream effects analysis (DEA), mechanistic networks (MN), and casual network analysis (CNA) prediction algorithms. Disease and function analysis were used to identify the disease and functional categories affected by FSCN1 depletion based on alteration in transcriptome data. The biological functions assigned to each network are ranked according to the significance of that biological function to the network [54].

### 4.7. BRCA TCGA Data Analysis

Gene expression data from BRCA TCGA data were retrieved from Xena Browser (https://xenabrowser.net/ accessed on 1 June 2021). Patients were divided into high and low according to median FSCN1 expression. Expression data were then subjected to the marker finder algorithm as described before [50,55].

### 4.8. Protein-Protein Interaction (PPI) Network

PPI network and Gene ontology (GO) analysis were conducted on FSCN1^high^ as well as genes downregulated in MDA-MB-231 FSCN1^KD^ model using STRING database https://string-db.org/ (accessed on 1 June 2021) as described before [56].

### 4.9. Survival Analysis of Human Breast Tumor Microarray Datasets

Breast cancer survival data were downloaded in accordance with Kaplan–Meier plotter portal Access Policies and were assessed using the Kaplan–Meier plotter portal website (https://kmplot.com/analysis/ accessed on 1 September 2021) [24] as previously described [21]. Briefly, the different genes in the tumor samples were categorized into high and low expression, and the survival curves of samples with high and low gene expression were compared by log-rank test.

### 4.10. Live Cell Assays

Quantitative kinetic measurements of cell migration, invasion, proliferation and adhesion assays were performed using CIM-plate 16 on xCELLigence Real Time Cell Analysis instrument, from ACEA Biosciences Inc. (San Diego, CA, USA) as previously described [21,22]. The xCELLigence software was used to acquire data and calculate the average migration, invasion, proliferation, and adhesion to collagen IV of all the replicates by increases in the slope (1/h) of the curve during 24 h. Graph displaying change in the slope between the different groups was generated.

## Data Availability

Processed data are provided as supplementary tables.

## Supplementary Materials

The following are available online at https://www.mdpi.com/article/10.3390/ph14121228/s1, Table S1. List of differentially expressed mRNA transcripts in FSCN1 knockdown vs control MDA-MB-231 cells (2.0 FC, *p*-value < 0.05). Table S2. List of differentially expressed miRNAs in FSCN1 knockdown vs control MDA-MB-231 cells (2.0 FC, *p*-value < 0.05).
